# Time-Domain ADC and Security Co-Design for SiP-Based Wireless SAW Sensor Readers

**DOI:** 10.3390/s25144308

**Published:** 2025-07-10

**Authors:** Zhen Mao, Bing Li, Linning Peng, Jinghe Wei

**Affiliations:** 1 School of Cyber Science and Engineering, Southeast University, Nanjing 210096, China; 101011865@seu.edu.cn; 2 China Electronics Technology Group Corporation No. 58 Research Institute, Wuxi 214072, China; pume1975_cnjs@sina.com

**Keywords:** surface acoustic wave, System in Package, wireless sensor, physically unclonable function, hardware security

## Abstract

The signal-processing architecture of passive surface acoustic wave (SAW) sensors presents significant implementation challenges due to its radar-like operational principle and the inherent complexity of discrete component-based hardware design. While System-in-Package (SiP) has demonstrated remarkable success in miniaturizing electronic systems for smartphones, automotive electronics, and IoT applications, its potential for revolutionizing SAW sensor interrogator design remains underexplored. This paper presents a novel architecture that synergistically combines time-domain ADC design with SiP-based miniaturization to achieve unprecedented simplification of SAW sensor readout systems. The proposed time-domain ADC incorporates an innovative delay chain calibration methodology that integrates physical unclonable function (PUF) principles during time-to-digital converter (TDC) characterization, enabling the simultaneous generation of unique system IDs. The experimental results demonstrate that the integrated security mechanism provides variable-length bit entropy for device authentication, and has a reliability of 97.56 and uniqueness of 49.43, with 53.28 uniformity, effectively addressing vulnerability concerns in distributed sensor networks. The proposed SiP is especially suitable for space-constrained IoT applications requiring robust physical-layer security. This work advances the state-of-the-art wireless sensor interfaces by demonstrating how time-domain signal processing and advanced packaging technologies can be co-optimized to address performance and security challenges in next-generation sensor systems.

## 1. Introduction

Passive SAW sensors have been widely adopted for precision measurements of diverse physical parameters, including but not limited to pressure and temperature [1,2,3,4], leveraging their unique electromechanical transduction properties. SAW sensors transduce physical quantities through variations in acoustic wave propagation characteristics, where environmental changes induce measurable shifts in time delay, phase coherence, or resonant frequency. This operating principle enables multiparameter detection by correlating perturbations in surface wave dynamics to targeted physical stimuli through distinct piezoelectric transduction mechanisms [5]. SAW sensors employ a tripartite architecture comprising a piezoelectric substrate, interdigital transducers (IDTs), and reflective grating arrays. The sensing mechanism bifurcates into two principal operational modalities: delay-line configurations exhibit parametric sensitivity manifested through temporal delay variations and phase distortion, whereas resonant-type implementations demonstrate frequency-selective responsiveness characterized by resonant frequency shifts [6]. These complementary transduction paradigms enable the realization of diverse wireless passive sensing systems tailored to specific measurement requirements.

While passive SAW sensors inherently benefit from their battery-less operation and structural simplicity, their reader systems face escalating design complexities to meet contemporary wireless sensing demands. The proliferation of SAW sensing technology has driven significant innovation in interrogator architectures and hardware implementation strategies. Kalinin [7] established a torque sensor based on a DSP-centric design at the processor clock 150 MHz with RF front-ends MLX11006, working at 433–437 MHz, allowing for a flat system frequency response. Gao [8] implemented a 920 MHz SAW reader receiving circuit leveraging an LT5516 IQ demodulator, ADF4350 local oscillator, and AD9288 dual-channel ADC with FPGA-controlled sampling. Pan [9] developed a 433 MHz gas sensor for dimethyl methylphosphonate (DMMP) detection, leveraging a reflective delay-line architecture incorporating single-phase unidirectional transducers (SPUDTs) and STM32 MCU-controlled frequency-stepped continuous-wave radar. Chen [10] implemented a 400 MHz high-speed data acquisition module (DAM) for SAW readers, employing RF bandpass sampling and digital quadrature demodulation to resolve time-delay and phase measurement challenges in large-capacity SAW tags.

As discussed above, ADC within DSP operates at only a few mega-samples per second, necessitating stringent design constraints on the front-end IF circuitry, or else the reader requires a dedicated high-speed ADC to perform analog-to-digital conversion of intermediate-frequency (IF) signals. Aforementioned SAW sensor readers generally exhibit a low level of integration and are large in size, making it increasingly difficult to meet the miniaturization demands of IoT applications. At the same time, to facilitate the measurement of multiple target sensors, we also need to distinguish and identify multiple reader IDs during measurement. To solve the problems mentioned above, joint design of time-domain ADCs and PUFs for SiP-based readers is proposed.

The major contributions of this paper are the following:

(1) In a SiP-based Multiprocessor System-on-Chip (MPSoC), this work presents the first implementation of a time-domain ADC in the programmable logic (PL) to process transient periodic echo signals from SAW sensors. Meanwhile, the processing system (PS) generates real-time graphical web browser interfaces that dynamically update the processed data. Compared to previous acquisition systems, this approach integrates sampling, signal processing, and visualization into a unified framework, eliminating the need for application in PC and dedicated hardware.
(2) The time-domain ADC eliminates the need for external ADC circuits and peripheral components. Additionally, SiP integration reduces the system volume and weight compared to the original system, achieving a more compact and lightweight design while maintaining functional integrity.
(3) Conventional IoT systems typically require additional hardware resources to enhance security. In contrast, this work integrates ADC sampling with a PUF challenge–response mechanism through a novel approach. During TDC calibration, the system exploits the inherent randomness of FPGA carry chain propagation delays to generate a unique hardware fingerprint. This method eliminates the need for extra hardware components while achieving seamless security integration.

The remainder of this paper is organized as follows. Section 2 presents the reader’s system architecture and operational principles, including the implementation of the SiP-based signal-processing unit and the working mechanism of the time-domain ADC. Section 3 elaborates on generating PUF stimuli and responses during the TDC calibration process. Section 4 analyzes the performance characteristics of the ADC and PUF, including their key metrics and evaluation methodologies. Finally, Section 5 summarizes this work’s key findings and contributions, while discussing potential applications and future research directions.

## 2. System Architecture

### 2.1. System Architecture and Work Process

**Figure 1:**
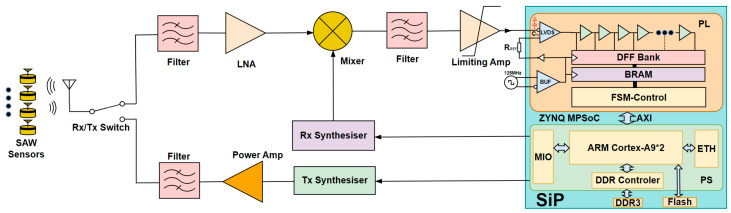
Passive SAW sensing signal reader system.

The SAW sensor system comprises a reader, an antenna, and a tag. The tag includes a tag antenna and a SAW resonator mounted on the target object’s surface. In our design, the reader operates in the time-division duplex (TDD) mode, transmitting a stepped single-carrier wireless signal within the 440 MHz ± 9 MHz frequency band for wireless interrogation. When a tag is within the measurement range, it responds by returning an RF signal at its resonant frequency $\left(f_{0}+k\Delta t\right)$. The reader calculates the current sensor value based on the frequency of the returned signal. Figure 1 illustrates the system architecture of the reader system.

After system initialization, the reader periodically transmits RF signals to interrogate the SAW sensors, with returned signals sampled at intervals. Upon receiving a sensor response, the reader employs an RF mixer to down-convert the signal to IF. The IF signal is directed to the LVDS input port, which is compared with a reference signal. This architecture leverages an ADC with a higher sampling frequency than prior designs using the DSP-embedded [7,11] or standalone external ADCs aforementioned, thereby reducing the performance demands on the front-end RF mixer and enabling direct RF sampling capabilities. The firmware within the programmable logic of the SiP then calculates the current sensor data using an algorithm and pre-calibration data. Subsequently, the PL aggregates the digital data into a structured format via the AXI bus. Data visualization is achieved through dual interfaces: the PL outputs a web user interface directly via HDMI, while the processing system generates a web-based graphical display. Remote terminals can access this web application for real-time visualization via Wi-Fi or ethernet interfaces.

### 2.2. Signal Analysis and Acquisition

The signal characteristics of the sensors were simulated and analyzed using MATLAB R2018b to optimize the performance of the reader’s signal-processing algorithm. A temperature sensor system was employed as a case study based on the detuning principle. Each SAW sensor exhibits a specific resonant frequency under given environmental conditions in this approach. Temperature variations induce corresponding shifts in this resonant frequency, which serves as the measurable parameter for temperature detection.

#### 2.2.1. Signal Characteristics at Resonance and Detuning

Within a single cycle, the excitation signal *x*(*t*), generated by the reader, activates the sensor. The signal is defined as follows:

(1) $$x(t)=a\cos(2\pi f_{0}t)$$

where *f*_0_ denotes the operating frequency and *a* represents the signal amplitude. When *f*_0_ matches the intrinsic resonant frequency of the SAW resonator, the system enters a state of oscillation, producing a reflected transient signal *y*(*t*):

(2) $$y(t)=A_{0}e^{-\frac{t}{\iota }}\cos(2\pi f_{0}t)$$

**Figure 2:**
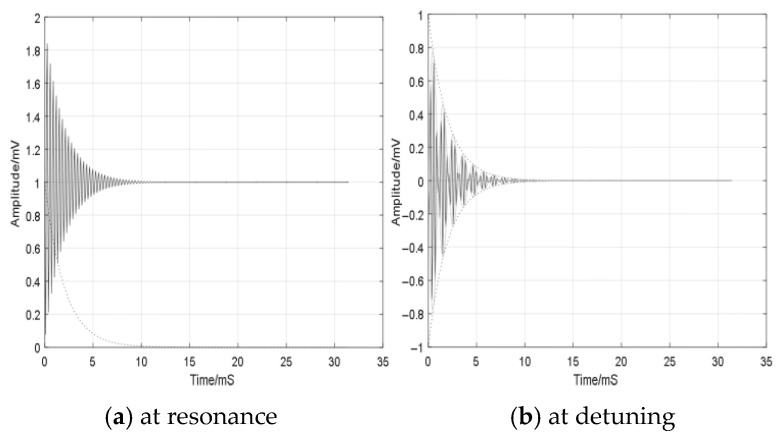
Transient response of a SAW sensor.

The signal exhibits a decay envelope denoted by *A*_0_$e^{\frac{-t}{\tau }}$, where the response profile is illustrated in Figure 2a. As depicted, the signal manifests as a bilateral band, operating at the same frequency as the active excitation signal, while its amplitude undergoes exponential decay over time. The transient response of the output signal is further presented in Figure 2b.

Variations in external temperature induce shifts in the resonant frequency. When the frequency of the emitted active signal deviates from the sensor’s resonant frequency by Δf, the resulting transient return signal is expressed as *y*(*t*):

(3) $$y(t)=A_{0}e^{-\frac{t}{\iota }}\cos(2\pi \Delta ft)\cos(2\pi f_{0}t+\varphi )$$

#### 2.2.2. Design of RF Transceiver Front-End

**Figure 3:**
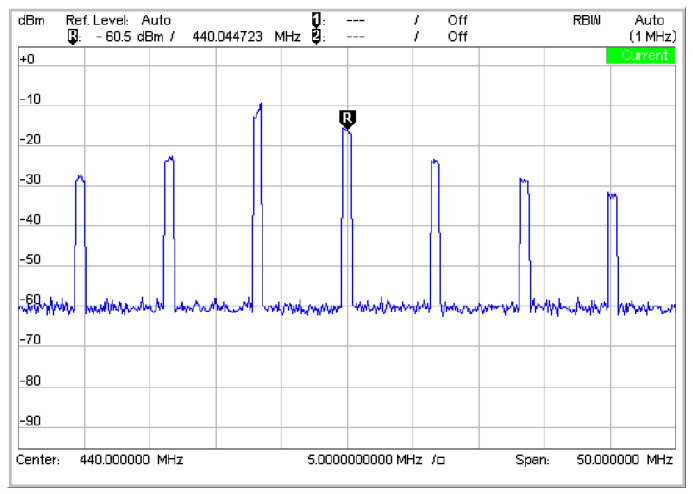
Sweep emission waveform with center frequency of 440 MHz.

The transmit path of the RF transceiver chip incorporates a Power Amplifier RFPA0133 to enhance output power. As shown in Figure 3, the frequency-swept transmit waveform at a center frequency of 440 MHz achieves an output power of approximately 10 dBm after signal processing through the PA and the high-speed RF switch PE42440. A low-noise amplifier is placed at the front of the receiving channel to reduce the noise factor level and amplify the signal. An RF bandpass filter suppresses the interference signal outside the working band. This filter has a center frequency of 440 MHz, a 3 dB bandwidth of 24 MHz, and 3 dB insertion loss [12].

**Figure 4:**
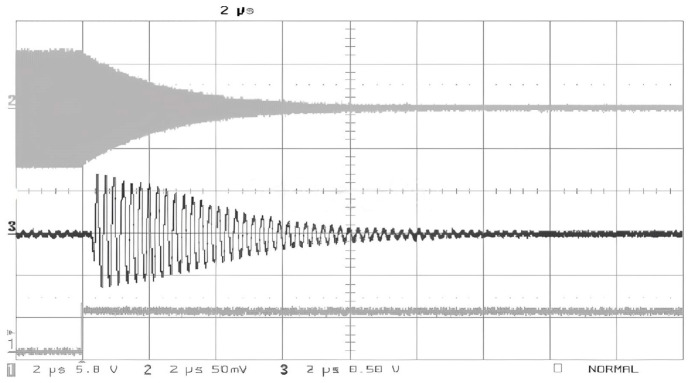
SAW tag reflection waveform.

Figure 4 illustrates the reflected signal waveform of the SAW tag. The upper decay curve represents the transmitted signal, the middle decay curve corresponds to the reflected signal, and the lower curve depicts the control signal waveform.

### 2.3. SiP Implementation

System-in-Package integrates multiple functional chips, such as processors and memory, within a single package to form a complete functional system [13,14,15]. Unlike System-on-Chip (SoC), which integrates components onto a single chip, SiP achieves system functionality by assembling different functional chips arranged in parallel or stacked configurations on a substrate before encapsulation.

The SiP design in this work follows a three-step fabrication process. First, to expand the bus width and increase cache capacity, two DDR3 dies are stacked on the substrate after redistribution layer (RDL) re-routing of their pads, followed by wire-bonding interconnects and encapsulation. Second, a Xilinx MPSoC series 7Z010 die, a wafer-level fan-out packaged Flash die, and the previously stacked DDR module are integrated onto a new substrate via secondary interconnects. DDR and flash components are mounted on the PS interface of the 7Z010 to form a minimal system configuration. Finally, a metal lid for thermal management and physical protection is attached to the integrated subsystem, while ball grid array (BGA) solder balls are placed on the substrate’s underside.

**Figure 5:**
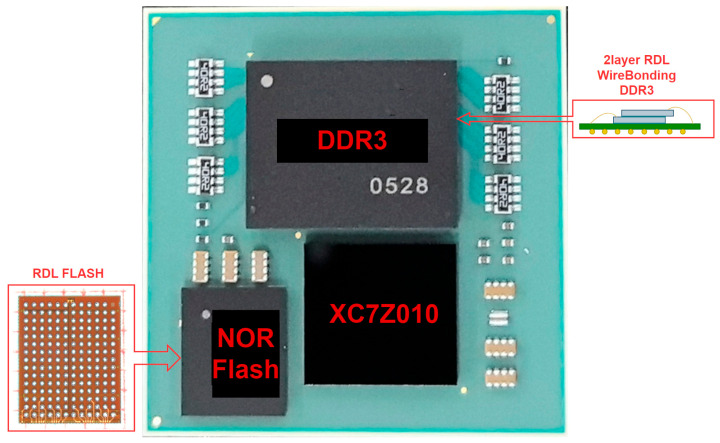
SAW sensor signal-processing critical subsystems based on SiP.

From a system-level perspective, SiP technology eliminates the area occupied by individual chip packaging by directly interconnecting dies on a substrate and packaging them. This reduces the spacing between dies and their interconnect distances. Furthermore, vertical stacking of multiple dies significantly minimizes the overall system footprint, enabling more compact designs. The completed SiP is illustrated in Figure 5, with dimensions of 23 mm × 23 mm × 5 mm. This integrated solution achieves approximately 30% space savings compared to conventional PCB interconnection approaches.

Previous SAW reader designs predominantly employed DSPs as the main controller. After collecting data from SAW tags, these systems required data transmission to a host computer via RS-485 interfaces, where system configuration and display interactions were handled through dedicated application software installed on the PC [7,8,9,10]. In contrast, the proposed system utilizes a 7Z010 SiP as the main controller. With DDR memory configured in PS, this architecture enables the direct implementation of a web server on the embedded Linux operating system running on the PS. Remote terminals can access the reader’s IP address through wired or wireless networks via standard web browsers, allowing for (1) real-time visualization of sensor data collected from multiple SAW tags, and (2) interactive configuration of system parameters through the web interface. Additionally, the system supports a direct visual output through HDMI in the PL of the SiP, providing a direct display when connected to monitors.

### 2.4. Time-Domain ADC Implementation in SiP

**Figure 6:**
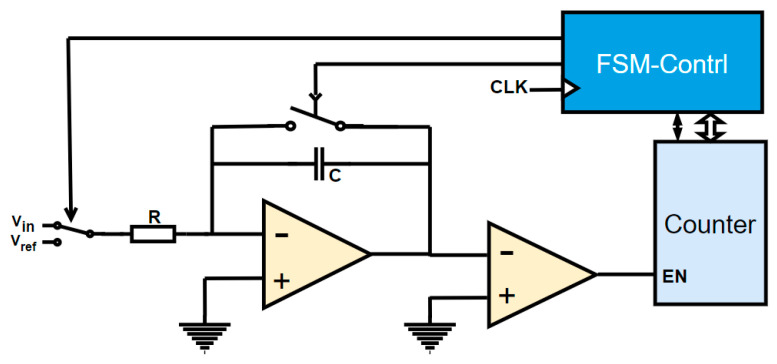
Double-integration ADC measurement schematic [16].

The classic time-domain ADC, also termed a dual-integration ADC, illustrated in Figure 6, is a foundational architecture for signal acquisition applications. This ADC comprises four core components: an operational amplifier, a comparator, a precision resistor, and a capacitor. Its operation hinges on two distinct integration phases to transform the input voltage into a proportional time interval, which is subsequently quantified by a counter to achieve analog-to-digital conversion.

The proposed time-domain ADC architecture introduces key improvements to enable flexible analog-to-digital conversion capabilities. Specifically, the system requires only an external resistor on the SiP pins to achieve an adjustable-resolution ADC with programmable sampling rates for signal acquisition.

In more advanced Ultrascale FPGA implementations, the external resistor is eliminated. By leveraging the FPGA’s internal programmable resistors and input capacitance, a 7-effective number of bits (ENOB) ADC operating at 600 Msps can be constructed directly within the digital front-end. This eliminates the need for external components while maintaining high-resolution signal acquisition at ultra-fast sampling rates [17].

**Figure 7:**
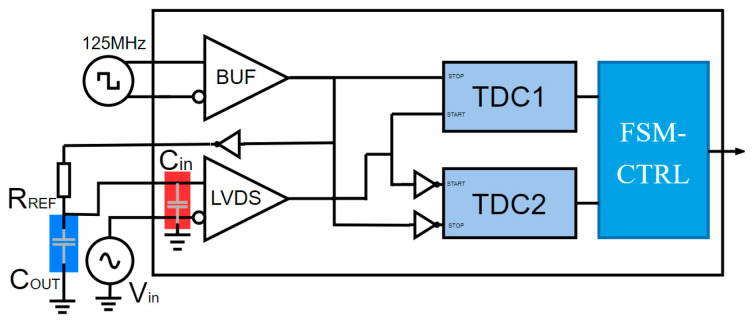
The block diagram of the time-domain ADC implemented in an FPGA.

Figure 7 illustrates the principal structure of the proposed analog-to-time-domain converter design. It fully leverages FPGA internal resources, including a high-speed low-voltage differential signaling (LVDS) receiver unit, a digital clock management unit, and two programmable delay lines based on the CARRY4 logic. After calibration, the time data conversion unit enables high-precision, high-speed analog-to-digital sampling with only an external resistor required to generate a reference voltage. When a comparator transition signal arrives, the carry chain propagation begins and continues until the rising edge of the clock arrives. At this point, the state of the carry chain is latched, yielding a digital code corresponding to the time difference between the transition occurrence and the clock edge.

The CARRY-based integrated time-domain ADC design draws inspiration from the principles of Δ-Σ ADCs [18,19]. Depending on peripheral component differences, capacitors can be placed parallel to the negative feedback input terminal and grounded, or the FPGA’s internal capacitors can be utilized.

The LVDS receiver acts as a comparator, illustrated in Figure 7, comparing the input analog signal with a reference voltage to generate high/low logic-level outputs. Three input signals are employed: a 125 MHz low-jitter clock source (with RMS jitter < 1 ps), and the two differential inputs of the LVDS buffer. The LVDS receiver outputs a high or low logic level after comparing the input analog signal with the reference voltage. Two TDC carry chains then measure the precise delay of these high/low signals within a single clock cycle. The FSM (Finite State Machine)-CTRL control module stores the measured data in block RAM (BRAM), which is subsequently read via the AXI bus for further processing.

The integrator circuit, coupled with resistor *R_REF_*, employs the LVDS input capacitance (*C_in_*) or an external capacitor (*C_out_*) connected to ground. The internal capacitance scheme (highlighted in the red box of Figure 7) is the most compact design, leveraging parasitic capacitance between the FPGA’s LVDS pins and the PCB pads (measured at ~50 pF). This configuration is non-invasive to the SiP’s functionality, as shown in Figure 7, where removal of the external resistor allows FPGA pins and resources to be fully reconfigured for standard operation.

When using *R_REF_* = 100 Ω, the integrator generates a rising slope of ~5 ns, exceeding half the clock period (20 ns at 50 MHz), ensuring complete ramp integrity. External capacitors (*C_out_*, shown in the blue box of Figure 7) offer a wider adjustment range but exhibit greater sensitivity to temperature fluctuations.

**Figure 8:**
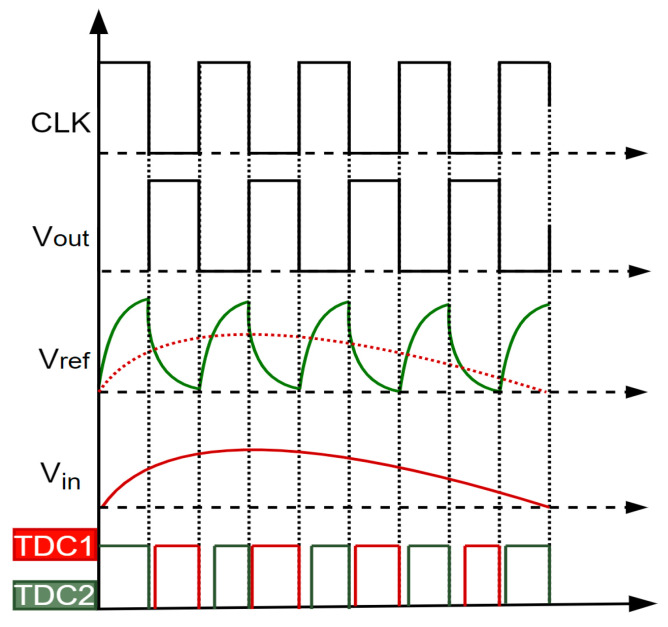
The timing waveforms of the system operation.

Figure 8 illustrates the system’s waveforms of operation. The design employs the SiT9120 differential active MEMS oscillator, which achieves a phase jitter (random) of 0.6 pS at 12 kHz–20 MHz (RMS). The rising edge of this clock signal acts as the STOP control signal for the carry chain TDC1, while its inverted edge provides the STOP control for TDC2. Additionally, the clock edge drives the input of the RC integrator circuit.

These time values are converted into digital codes for output. At the start of each clock period, the reference voltage begins rising. When the input voltage (*V*_in_) exceeds the reference voltage (*V*_ref_), the comparator output goes high, and TDC CH0 records the time difference between this transition and the clock’s rising edge. Subsequently, as the reference voltage declines, *V_in_* falling below *V_ref_* triggers a low comparator output, which TDC CH1 measures. This process yields two time measurements per clock cycle, effectively converting two analog input signal samples into digital values.

The TDC architecture consists of components including lookup tables (LUTs), carry chains, D flip-flop (DFF) triggers, a ring oscillator for calibration, an encoder, and block RAM (BRAM). The carry chain, a fundamental internal structure in FPGAs, experiences propagation delay variations in each unit due to process, voltage, and temperature (PVT) effects. These variations lead to non-uniform propagation delays through the delay chain, preventing consistent step increments. Consequently, prior calibration is essential to enhance the carry chain’s measurement accuracy in practical applications [20,21].

**Figure 9:**
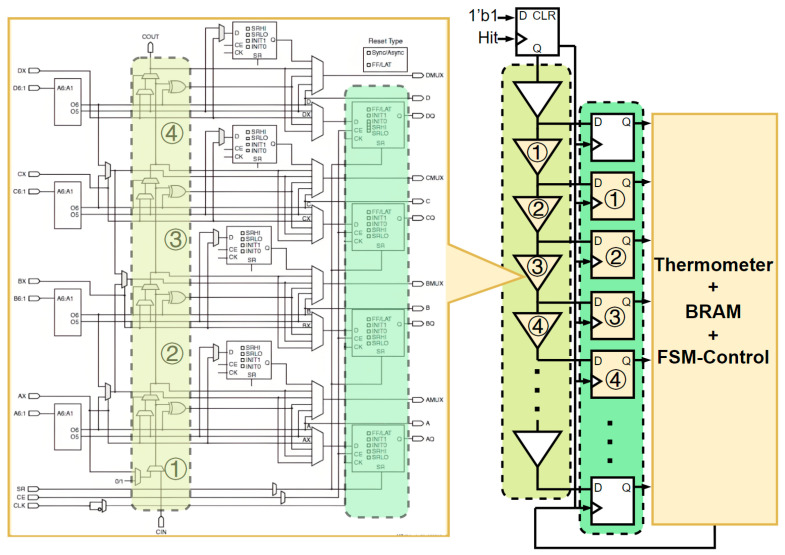
Schematic of the carry chain within the SLICEM.

As illustrated in Figure 9, the structural diagram of the SLICEM unit reveals that each SLICE contains four interconnected carry units. Each carry unit’s COUT (carry-out) is linked to the CIN (carry-in) of an adjacent SLICE’s carry unit, forming a cascading chain. Furthermore, the output of every carry unit is coupled with a D flip-flop (DFF) within the SLICE. This unique architecture enables the construction of extended-length carry chains by serially connecting multiple CARRY units.

During TDC operation, the Hit signal enters the first carry unit in the chain and propagates sequentially through each carry unit. The arrival time at each carry unit is captured and recorded by the corresponding DFF. This process continues until a second Hit signal arrives. The recorded DFF data indicates the number of carry units traversed by the signal between the START and STOP events. Since each carry unit’s propagation delay is pre-calibrated, the recorded position data enables precise calculation of the total propagation delay through the chain. Figure 9 illustrates the TDC’s internal structure, which requires calibration of the delay for every carry unit in the first column before deployment.

This design employs a code density analysis method to calibrate individual carry units and their interconnections to achieve self-testing objectives while minimizing test costs. The calibration framework is rooted in coherent sampling theory, establishing a frequency difference between the TDC’s main clock and the calibration signal. This frequency offset generates pseudorandom signal arrivals, ensuring a uniform distribution across the TDC’s delay path and mitigating systematic errors.

A ring oscillator is embedded within the FPGA to generate oscillation-based calibration pulses. The oscillator’s frequency must be intentionally non-correlated with the TDC’s main clock to randomize the arrival times of calibration signals at each carry unit. This non-correlation ensures that delay measurements are statistically independent, thereby enhancing the robustness of the calibration process against PVT-induced variations.

## 3. Delay Line Calibration and PUF Design in SiP

### 3.1. Code Density Analysis for Calibration

The code density analysis method solves mathematical or physical problems through random sampling approximation and is widely applied in numerical integration and probability density estimation. Based on its statistical analysis principles, when a sufficient number of random excitation pulses enter the carry chain, the count of pulses falling within the time width of a delay unit is directly proportional to that unit’s delay time.

**Figure 10:**
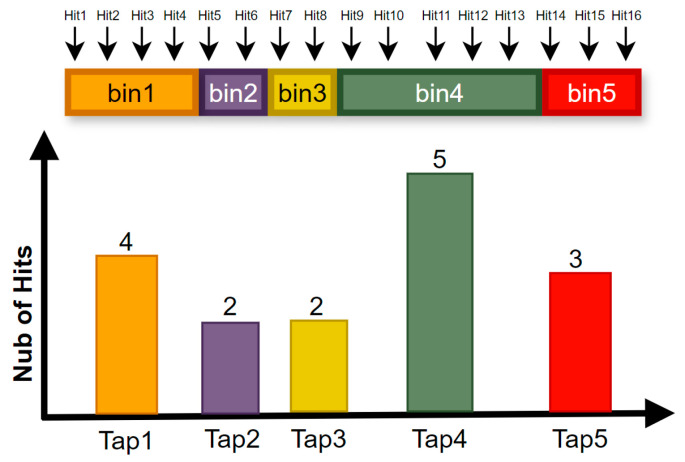
Schematic of the code density analysis method for calibration.

The code density analysis employs a statistical calibration methodology that leverages the random distribution of calibration signals. By analyzing the frequency at which each code is triggered, the algorithm estimates the actual time width of each carry unit within the TDC, as illustrated in Figure 10. Specifically, during a single clock cycle, a large number of random pulse edges enter the TDC delay chain and are distributed across the time intervals defined by the propagation delays of individual delay units. The accumulated count of pulse edges captured by each delay unit is integrated to determine its time width. Once all delay units’ time widths along the TDC chain are quantified, these values are stored in a lookup table (LUT) for subsequent calibration purposes.

As previously noted, the calibration hitting pulses are treated as random signals relative to the TDC clock. The relationship between the standard deviation and the number of calibration pulses N is defined in Equation (4), T is the TDC main clock period, K denotes the number of delay taps in the delay chain, $\sigma_{m}$ represents the maximum standard error, and $\sigma_{i}$ is the standard deviation of the i-th delay unit. This equation quantifies how calibration precision improves with increasing N, while accounting for statistical uncertainties across the delay chain.

(4) $$\sigma_{m}=\frac{Tc}{N}\sqrt{\sum_{i=1}^{K}\;\sigma_{i}^{2}+\frac{\sigma_{i}^{2}}{2}}<\frac{Tc}{\sqrt{N}}\sqrt{1-\frac{1}{K}}<\frac{Tc}{\sqrt{N}}$$

**Figure 11:**
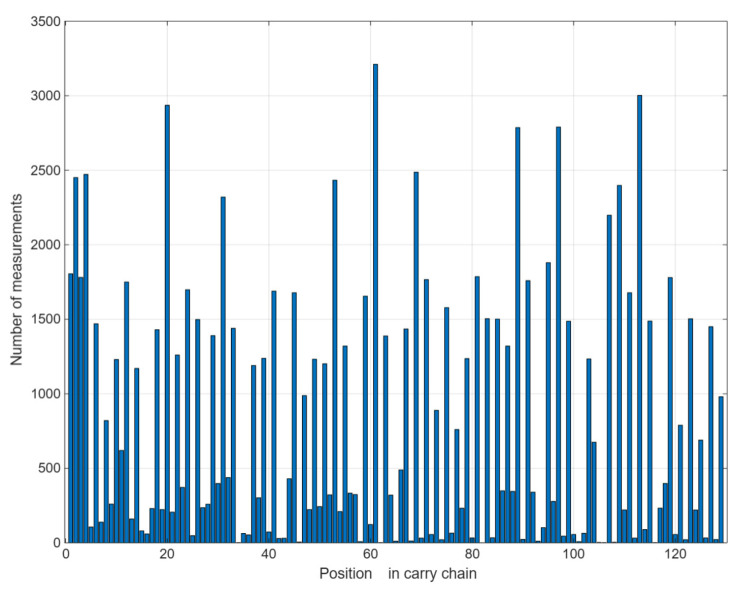
Code density test results (before calibration).

When the TDC clock period *Tc* is set to 8 ns and the required standard deviation $\sigma_{m}$ must be less than 5 ps, the number of calibration random hitting pulses must exceed 2,560,000. Figure 11 displays the first 129 bins of 452 taps, representing the raw measurement data before calibration in the rising edge.

Following calibration of the TDC carry chain, extensive statistical analysis was conducted on the programmable delay units IDELAY within Xilinx FPGA’s IOBLOCK through repeated measurements. The standard deviation σ was used to evaluate the calibrated TDC delay chain’s accuracy and stability. Concurrently, the resolution of the CARRY-based TDC delay chain for picosecond-level delays was further validated by incrementally adjusting the IDELAY tap values and comparing the resulting measurements. The standard deviation σ was calculated using Equation (5), where *N* represents the number of repeated measurements, and $x_{j}$ corresponds to the measured delay value of the *j*-th tap.

When the IDELAY tap value was set to 0, the averaged delay was approximately 656 ps. For a fixed IDELAY tap value, the standard deviation σ calculated via Equation (5) was approximately 2.3. This result demonstrates that the calibrated TDC delay chain can accurately and reliably measure propagation delays in both on-chip and off-chip environments.

(5) $$\sigma =\sqrt{\frac{1}{N}\sum_{i=1}^{N}\;{\left(x_{i}-\frac{1}{N}\sum_{j=1}^{N}\;x_{j}\right)}^{2}}$$

**Figure 12:**
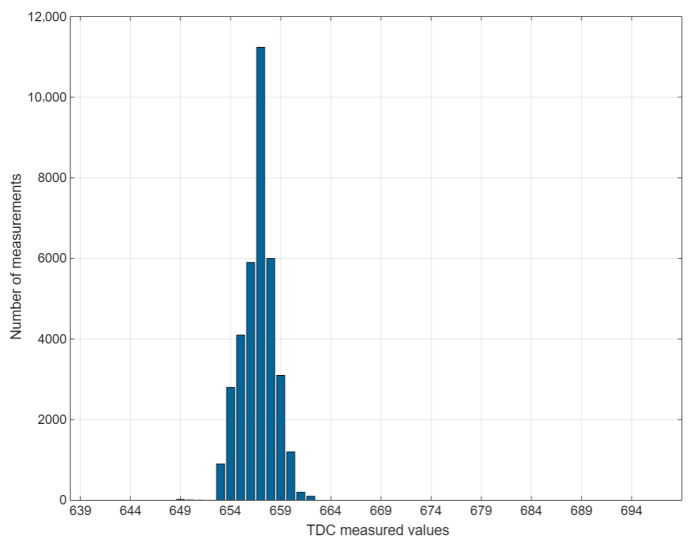
TDC measurement results for IDELAY’s propagation delay.

The collected measurement data were graphically analyzed, as illustrated in Figure 12. The analysis revealed that the measured values conform to a Gaussian distribution with the highest probability density between 654 ps and 657 ps. When the IDELAY tap value was incremented, the center of the probability density distribution shifted rightward by 74 ps ± 21 ps, accompanied by minor fluctuations in the data. Post-calibration ADC nonlinearity test results: INL: −0.98 to 0.86 LSB, DNL: −0.79 to 0.84 LSB at 125 MSample/s. The ADC measurement range is 0.37 V to 2.10 V. As the measured voltage increases, the time delay required to be measured by the ADC proportionally extends.

**Figure 13:**
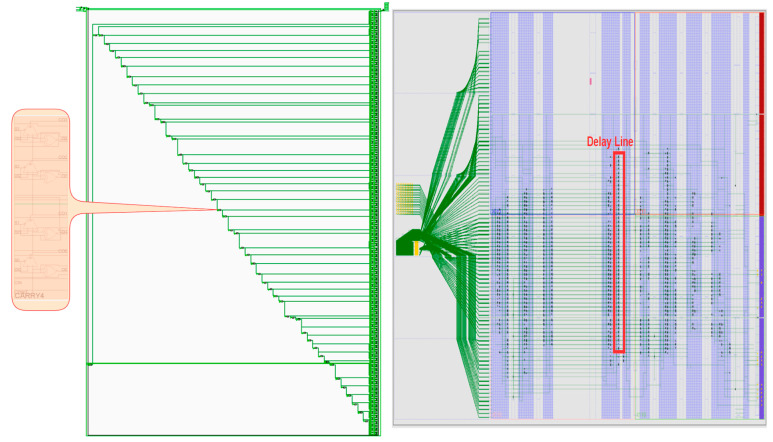
Schematic of the synthesized delay chain and post-layout distribution of physical units.

When implemented on the Xilinx Zynq 7Z010, San Jose, CA, USA, it is essential to ensure that the total time width of the TDC delay chain exceeds the single cycle time of the TDC main clock. The TDC delay chain length is set to 452 taps, as illustrated in Figure 13. Due to the unique structural characteristics of the CARRY, the delay chain unit count is defined in the Verilog description by cyclically invoking hardware primitives. During the Vivado implementation phase, the tool automatically concatenates one stage’s COUT (carry output) with the subsequent stage’s CIN (carry input), forming the delay chain.

In the Verilog code for generating the delay chain, the number of delay units can be adjusted by simply modifying the STAGE parameter. However, while synthesis tools (e.g., Vivado 2019) can automatically concatenate the delay chain, careful consideration must be given to its length and physical placement within the FPGA fabric. Post-implementation validation is also required to ensure the layout and routing of the delay chain are logically consistent.

**Figure 14:**
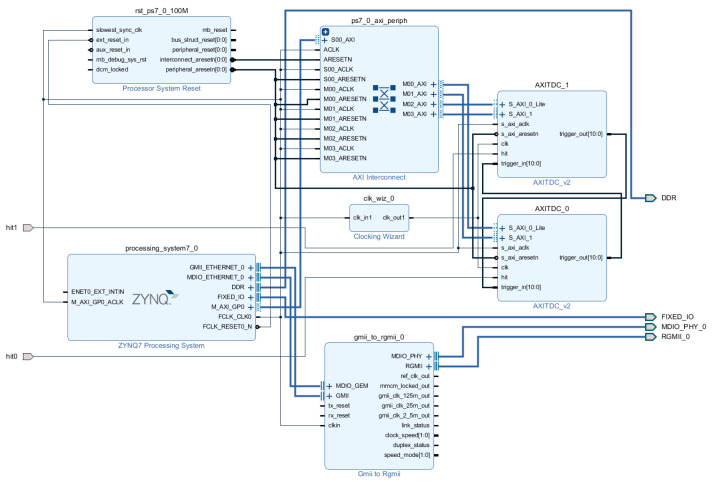
Block diagram of the TDC design.

The delay chain, composed of DFF triggers interconnected via CARRY4 blocks in the CLB (Configurable Logic Block), generates a binary sequence in the form of “****11110000****”. This bitstream’s length corresponds to the number of delay units and must be converted into an 8-bit binary encoding for transmission. The block diagram of the Vivado project is depicted in Figure 14. Due to the RGMII interface of the Ethernet PHY chip on the experimental board, a GMII_to_RGMII_0 IP core was integrated to perform bus-width conversion. The hit0 and hit1 signals are routed to the AXITDC_0 and AXITDC_1 modules, respectively. The processing_system7_0 (PS) module is primarily configured for clock input management, ethernet interface control, and DDR memory controller setup.

### 3.2. PUF Implementation While Calibrating

As discussed in Section 3.1, the code density test yields a one-dimensional array in which each element corresponds to the propagation delay of a carry chain unit. Due to process variations, these delays exhibit random characteristics. Based on this property, a segmentation method can be devised to convert the one-dimensional array into a random binary sequence using a binarization method. The generated binary sequence should ensure a uniform distribution and alternation of 1 s and 0 s. Below, we analyze and compare binarization segmentation methods using experimental data.

#### Binarization Segmentation Methods

1. Median Segmentation

Each element in the array is compared to the median of the entire array. If the element is greater than the median, it is assigned a value of 1; otherwise, it is 0. While straightforward, this method may lead to uneven distributions if the data exhibits trends (e.g., a gradual increase or decrease), resulting in a consecutive 0 s or 1 s. However, median segmentation is appropriate if the data is randomly distributed.

2. Regression Curve Fitting

A regression curve (e.g., a second-order polynomial) is fitted to the data. Elements above the curve are assigned 1, while those below are assigned 0, generating a binary sequence. This method outperforms median segmentation and differential encoding when the data fluctuates around an ascending or descending trend, offering broader applicability.

3. Moving Average Filtering

A moving average is used as a threshold, where each element is compared to the average of the preceding k elements (e.g., k = 5). If the current element exceeds this average, it is assigned 1; otherwise, it is 0. When the data oscillates around the moving average, the resulting binary sequence exhibits frequent alternations between 1 and 0.

4. Differential Encoding

The difference between adjacent elements is computed, with positive differences encoded as 1 and negative differences as 0. This method produces a rapidly alternating binary sequence if the data fluctuates frequently. However, if the data exhibits monotonic trends (continuously increasing or decreasing), it may generate long sequences of consecutive 1 s or 0 s.

5. Quantile-Based Segmentation

The array is dynamically divided into segments (e.g., of length 10), and a local threshold (median or mean) is applied within each segment to generate the binary sequence. This approach adapts to regional variations in the data, improving uniformity. For the given dataset, varying the segment length had minimal impact on the binary sequence.

**Figure 15:**
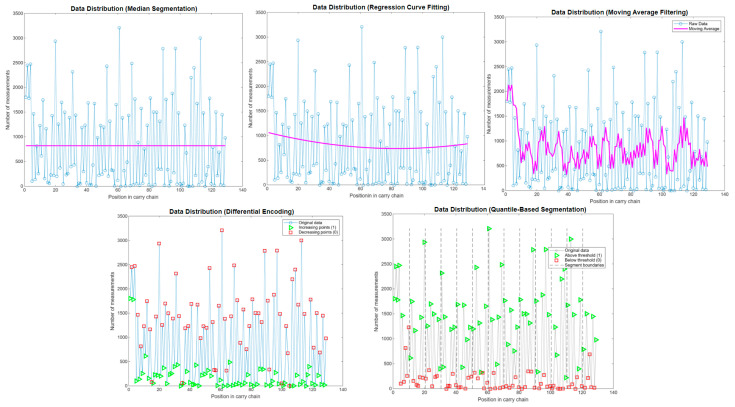
Comparison of binarization segmentation algorithms.

Figure 15 illustrates the binarized sequences generated by the above algorithms. For the given dataset, median segmentation, regression curve fitting, and quantile-based segmentation yield similar results, with comparable uniformity and alternation of 1 s and 0 s.

Table 1 presents the binary sequences generated through binary segmentation of the array using the five aforementioned algorithms. For Regression Curve Fitting, a second-order polynomial was employed in the regression curve fitting process. Moving Average Filtering utilized a moving average window size of 5, with adjustable window dimensions based on specific requirements. In Quantile-Based Segmentation, the segmentation length was set to 10; however, for this particular array, variations in segmentation length produced minimal impact on the resulting binary sequences. Comparative analysis indicates that Regression Curve Fitting demonstrates superior applicability. Higher polynomial orders in Regression Curve Fitting yield regression curves that more closely approximate the data distribution, enhancing both the interval regularity and uniformity of the data distribution. However, increased polynomial orders also introduce certain bit instability phenomena [22]. Specifically, identical carry chains may produce inconsistent binary outputs when repeatedly processed with the same polynomial curve fitting algorithm, resulting in bit flipping at specific positions. This demonstrates an inverse relationship between polynomial order and bit stability—higher polynomial orders correspond to reduced bit stability.

## 4. Results

After calibration, the TDC is employed to implement a time-domain ADC, which significantly enhances the ADC’s performance. During the TDC calibration process, a binary sequence is generated by leveraging the characteristics and implementation principles of PUF. This sequence can be a SiP identification code or an embedded root cryptographic key.

### 4.1. ADC Performance and Effective Number of Bits

**Figure 16:**
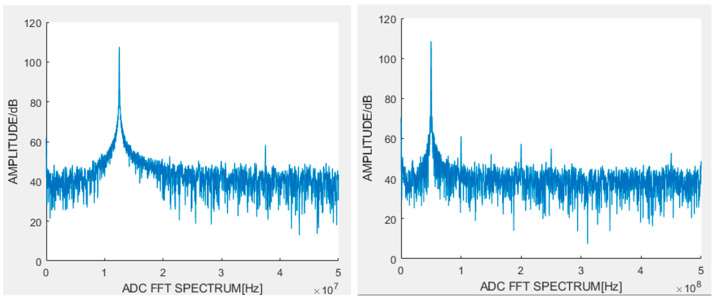
The time-domain ADC samples the spectrum of a sinusoidal signal with frequencies of 12.5 MHz and 50 MHz.

A time-domain ADC’s performance can be evaluated by measuring its signal-to-noise-and-distortion ratio (SNDR) and effective number of bits (ENOB). This experiment generated sinusoidal signals with frequencies of 12.5 MHz and 50 MHz (peak-to-peak amplitude of 2.5 V) as test inputs. The output samples from the time-domain ADC were analyzed using Fast Fourier Transform (FFT), with the resulting spectra for both frequencies shown in Figure 16.

Experimental results demonstrate that for the 12.5 MHz input signal, the measured SNDR is approximately 39 dB.

(6) $$ENOB=\frac{SNDR-1.76}{6.02}$$

This corresponds to an ENOB of about 6.2 bits. Similarly, for the 50 MHz input signal, the measured SNDR is approximately 36 dB, yielding an ENOB of roughly 5.8 bits.

### 4.2. PUF Characteristic Analysis Results

The binary sequence generation methodology in Section Binarization Segmentation Methods employs regression curve fitting segmentation. The subsequent PUF characteristic analysis presented in the following section is based on the binary sequence sets of 30 saw sensor reader boards.

#### 4.2.1. Reliability Results

PUF reliability refers to consistently producing the same output within an error margin under repeated identical stimulations (intra Hamming distance). The reliability evaluation of the PUF is conducted as follows: For a given challenge *c_i_*, the reference response is recorded as an n-bit value. Subsequently, *m* repeated response measurements (*R_i_*_1_, *R_i_*_2_, …, *R_im_*) are collected using the identical challenge *c_i_* The PUF’s reliability metric is then computed by statistically analyzing the intra-chip Hamming distances between each measured response and the reference response *R_i_* This methodology quantitatively assesses the PUF’s response consistency under identical excitation conditions.

(7) $${HD}_{\mathrm{INTRA}\;}=\frac{1}{m}\sum_{t=1}^{m}\;\frac{HD\left(R_{i},R_{i,t}^{\prime }\right)}{n}\times 100\%$$

In the temperature stability test, response bits from a single chip were measured at varying temperatures, and the resulting Hamming distance was calculated. The built-in temperature diode of XC7Z010 is used to accurately measure the chip’s temperature. The chip’s temperature is continuously monitored using the Chip-Scope tool in Vivado, and the temperature value is recorded during bit-string generation. Measurements of circuit stability were taken at ten different temperatures between 10 °C and 75 °C, with ten samples collected at each temperature. The reliability of the PUF decreases slightly with increasing temperature after PUF generation at 25 °C, reaching 97.56.

#### 4.2.2. Uniformity Results

The uniformity of PUF serves to quantify the distribution characteristics of PUF responses. Theoretically, the binary responses (0 and 1 bits) should follow an equiprobable distribution. Furthermore, individual response bits must maintain mutual independence without correlation, ensuring that adversaries cannot predict unknown response values based on known challenge–response pairs (CRPs).

(8) $$(Uniformity{)}_{i}=\frac{1}{n}\sum_{l=1}^{n}\;r_{i,l}\times 100\%$$

The uniformity metric is calculated as shown in Equation (8), where *r* represents an n-bit PUF response, and $r_{i,l}$ denotes the l-th bit value of the response. The uniformity result is 53.28.

#### 4.2.3. Uniqueness Results

PUF uniqueness refers to the capability of uniquely identifying a specific integrated circuit among a population of nominally identical chips. This property is quantitatively evaluated by measuring the Hamming distance between the response pairs generated from different PUF instances under identical challenge conditions. The evaluation methodology involves the following steps: (1) Apply identical challenge inputs to two distinct PUF hardware instances. (2) Extract their corresponding response outputs. (3) Compute the inter-chip Hamming distance (HD) between the response pairs. The uniqueness result 49.43 is calculated using Equation (3).

(9) $$\mathrm{Uniqueness}\;=\frac{2}{k(k-1)}\sum_{i=1}^{k-1}\;\sum_{j=i+1}^{k}\;\frac{HD\left(R_{i},R_{j}\right)}{n}\times 100\%$$

where *k* represents the number of quantized responses, *n* denotes the response sequence length, and $HD\left(R_{i},R_{j}\right)$ corresponds to the inter-chip Hamming distance between two PUF responses.

As illustrated in Table 2, the proposed design exhibits a significantly lower total slice count than state-of-the-art PUFs. Furthermore, the incremental rise in slice resource utilization remains marginal even with increasing response bits. Notably, the reliability and uniqueness metrics closely approach ideal theoretical values, demonstrating a robust performance across key evaluation criteria.

## 5. Discussion

As presented in this work, the operational principle of the time-domain ADC implemented in SiP shares conceptual similarities with sampling oscilloscope techniques. This architecture proves particularly suitable for SAW signal processing since only frequency extraction from waveforms is required, and the time-domain ADC demonstrates an excellent performance in analyzing periodic or repetitive signals.

The proposed design offers significant advantages for multi-sensor applications where a single reader interfaces with multiple SAW sensors. The inherent structure of time-domain ADC facilitates efficient implementation of multi-channel signal acquisition within FPGA architectures, making it ideal for such one-to-many signal collection scenarios.

SiP integration could enable the time-domain ADC to perform signal acquisition and BIST. Beyond external signal collection, the architecture could be extended to monitor internal parameters, including voltage fluctuations, signal jitter, and temperature variations. As proposed in this study, the integrated approach combining signal acquisition with PUF design exhibits broad applicability. This methodology can be extended beyond SAW readers to applications requiring signal acquisition capabilities and hardware security features.

## Figures and Tables

**Table 1:** Comparison of the binarized sequences generated by the five algorithms.

Data Set	1805,2451,1781,2472,106,1473,139,821,260,1233,624,1753,162,1175,82,61,235,1432,223,2936,206,1261,372,1698,48,1498,236,259,1392,398,2324,438,1439,2,63,53,1190,302,1238,73,1689,29,31,430,1678,5,988,223,1232,243,1201,322,2433,217,1326,333,324,8,1655,123,3211,2,1388,320,11,489,1435,12,2487,32,1766,56,889,21,1578,65,761,232,1236,33,1786,1,1504,34,1501,349,1320,345,2786,23,1759,340,11,102,1880,278,2790,45,1487,56,8,64,1234,675,3,2,2198,4,2397,221,1676,32,3002,89,1488,2,233,398,1780,56,789,21,1503,224,689,33,1453,22,981
Median Segmentation	111101000101010001010101010010101000101010001010101010100010101000101010101000101010101010100010101000100010101010100010001000101
Regression Curve Fitting ^1^	111101000101010001010101010010101000101010001010101010100010101000101010101010101010101010100010101000100010101010100010001000101
Moving Average Filtering ^2^	010100000101010001010101010010101000101010001010101010100010101000101010101010101010101010100010101000110010101010100010101010101
Differential Encoding	10101010101010011010101010110101010101010111010101010100010101001101010101010101010101010100110101001100010101010101110101010101
Quantile-Based Segmentation ^3^	111101000011010001010101010011111000101010011010101010110010101001101010101010101010101010110010101000110010111010100110101000101

^1^ Second-order polynomial. ^2^ The preceding elements k = 5. ^3^ The segment’s length is 10.

**Table 2:** Comparison of experimental results and data.

PUF	Uniformity	Uniqueness	Reliability	Response. Bit Length	Total Slices	Target FPGA
Anandakuma et al. [23]	49.48	48.38	99.94	256	93	Artix-7
Zhang et al. [24]	49.5	49.33	95.45	136	186	Virtex-5
Yan et al. [25]	50.05	N/A *	99.33	128	647	Artix-7
CARRYs (Our work)	53.28	49.43	97.56	128 **	32	ZYNQ-7000

* N/A means that this parameter is not mentioned in the reference. ** Response length configured for this work, actual variable.

## Data Availability

The original contributions presented in this study are included in the article. Further inquiries can be directed to the corresponding authors.
